# The impact of public transportation on the transmission of COVID-19 in Rwanda

**DOI:** 10.3389/fpubh.2024.1345433

**Published:** 2024-02-27

**Authors:** Brigitte Umutoni, Jean Claude Tumushime, Benjamin Hewins, Jean Claude Udahemuka, Pacifique Ndishimye, David J. Kelvin, Gustavo Sganzerla Martinez

**Affiliations:** ^1^College of Medicine and Health Sciences, University of Rwanda, Kigali, Rwanda; ^2^Center for Research and Innovation, African Institute for Mathematical Sciences (AIMS), Kigali, Rwanda; ^3^Department of Microbiology and Immunology, Dalhousie University, Halifax, NS, Canada; ^4^Department of Pediatrics, Izaak Walton Killam (IWK) Health Center, Canadian Center for Vaccinology (CCfV), Halifax, NS, Canada; ^5^Department of Veterinary Medicine, University of Rwanda, Nyagatare, Rwanda

**Keywords:** COVID-19, SARS-CoV-2, public transportation, epidemiological trends, disease transmission

## Abstract

**Introduction:**

The onset of the COVID-19 pandemic has placed a significant burden on healthcare systems worldwide, particularly in sub-Saharan regions where healthcare resources are limited. The transmission of SARS-CoV-2 is facilitated by the movement of people from place to place. Therefore, implementing measures that restrict movement of people and contacts is crucial in controlling the spread of the disease. Following the identification of the first COVID-19 case in Rwanda, the government implemented stringent measures, including a complete nationwide lockdown, border closures, curfews, reduced capacity in public transportation and businesses, and mandatory testing. This study aims to assess epidemiological trends in COVID-19 cases in relation to changes in population mobility within the public transportation system.

**Methods:**

A descriptive analysis using publicly available data on COVID-19 epidemiological indicators (cases, deaths, vaccinations, and stringency index) and mobility data was conducted.

**Results:**

The results reveal a strong correlation between mobility in public transportation and other activities, underscoring Rwanda’s reliance on its public transportation system. The study also identifies a pattern where increases in transit station mobility preceded spikes in COVID-19 cases, suggesting that the subsequent rise in public transportation usage may contribute to higher infection rates.

**Discussion:**

Therefore, this study emphasizes the importance of ongoing vigilance and regulatory measures regarding public transportation during infectious disease outbreaks.

## 1 Introduction

Rwanda is a small, landlocked country located in of East Africa. Rwanda shares a border with several East African Community (EAC) member states including Democratic Republic of Congo (DRC), Uganda, Tanzania, and Burundi. The location of Rwanda within the EAC has made it an important hub for trade and commerce within the region. Rwanda has achieved impressive economic growth in recent years, with an annual gross domestic product (GDP) growth rate of approximately 10.9% by 2021 (1). The transportation sector, for example, has grown rapidly with an annual vehicle growth rate of 12%. As of 2020, the number of registered vehicles countrywide rose to 221,000, consisting of 52% motorcycles and 38% passenger busses, of which at least 30,000 are in Kigali, the capital of Rwanda (2). The global evolution of transportation and population mobility has facilitated more rapid movement of people and goods, increased accessibility to jobs and services, and promotes economic growth. This advancement has led to high population densities in urban areas and increased exposure to infectious diseases (3), including SARS-CoV-2. SARS-CoV-2, the coronavirus responsible for the COVID-19 pandemic that emerged in China in late 2019, and in a short period, caused significant global burden due to its rapid spread via human-to-human contact and airborne transmission.

The spread of infectious diseases is promoted by people moving from place to place. As an example, the spread of the Black Death (*Yersinia pestis*) in Europe may have been due to the continuous movement of people and exchanging of goods in the Indo-Iranian silk road, which were Eurasian trade routes that spanned from China to Italy in the mid-15^th^ century (4, 5). Moreover, recent studies have linked the impact of severe events such as the spread of SARS-CoV-2 with the mobility of people. Evidence has shown that periods of higher disease transmission and introductory events follow seasonal patterns related to individual movement patterns within a population (6–10). Thus, a method of controlling the spread of infections is achieved through the implementation of measures that restrict contact between individuals. For instance, physical distancing policies have been implemented by governments to reduce and restrict social contacts in the population, such as school closure, workplace closure, bans on public events, and requirements to stay at home (11–14).

Since the onset of the COVID-19 pandemic, there have been numerous studies conducted to investigate the link between governmental reduced population mobility and viral transmission using a variety of methods (15–17). Globally, public health authorities implemented a wide range of transmission mitigation measures during early stages of the pandemic to control the spread of disease, including reduced social interaction, social distancing measures, masking policies, reduced public transportation, required isolation, and recommended hygiene practices, among others. Public adherence to these restrictions and governmental stringency are both implicated in determining the level of containment throughout the pandemic (10, 17, 18). Mobility research has largely focussed on the specific risks assigned to individual activities, such as school, work, leisure/travel, and interactions in these settings (19). Importantly, a better understanding of the interface between population mobility and infectious diseases in an urban setting is needed.

The role of urban infrastructure and planning has been explored in the context of COVID-19 transmission and suggests that transmission risk is heightened by particular transportation modes. For example, travel via air flights or high-speed rail from Wuhan was associated with a greater number of COVID-19 infections in the destination (20, 21). A study in Norway determined that those who tested positive for SARS-CoV-2 (prior to lockdowns) were 1–3 times more likely to have previously used public transportation (OR = 1.5), such as busses, trams, ferries, or trains (22). Alternatively, methods of transportation such as walking, or cycling are resilient to pandemics and represent low-cost alternatives to reducing pressure on crowded transportation systems. Facilitating the ease of pedestrian and cyclist transportation during urban planning therefore serves as a preventative measure for future pandemic preparedness. In underrepresented cities, or those situated in low-middle income countries (LMICs), such urban planning is heavily restricted by available resources. Indeed, individuals in these areas rely heavily on often crowded public transportation networks.

Studies exploring urban mobility in LMICs are scarce and necessitate greater research investment in these areas, especially given the heavy reliance on public transportation in these cities. One study in Bogotá, Columbia, found a reduced volume of passengers as well as reduced overall levels of the number of connections between bus stops during early lockdown stages of the COVID-19 pandemic (23). Interestingly, the same study found that those in a lower socioeconomic bracket exhibited higher levels of mobility during early stages of the pandemic. A separate study compared mobility trends between lower-and upper-income counties in the United States during the COVID-19 pandemic and found that individuals in rich counties were more responsive to restrictions (lowering mobility) compared to poorer counties (24). According to data collected in 2017 (25), motorcycles composed 51.09% of all the vehicles registered in Rwanda. Moreover, motorcycles were found to be the most predominant vehicle in a vehicle composition survey in 12 different locations of Kigali. Among the categories of motorcycles, passenger cars, minibusses, medium busses, busses, light good vehicles, and heavy good vehicles, motorcycles alone accounted for 52.8% of the traffic volume at different locations. According to the report, the most common methods of public transportation in Kigali are mototaxis and busses, whose purpose is for people to go to work (58%) or do business (17%). It is worth mentioning that in Kigali, common areas, such as bus terminals, are used as public transport hubs. From these hubs, people hire mototaxis or use busses to reach their final destinations. Moreover, the same report found that people who use the public transport in Kigali, do it at least three times a week. Finally, the majority of public transport users in Kigali earn an income from 0 to 100,000 Rwandan francs, the lowest category present in the survey. In this case, the working class of Kigali, a representative of its entire population, is heavily dependent on public transportation, which appears as mototaxis and busses.

Prior to the emergence of COVID-19 in Rwanda, the country had experience and knowledge regarding Ebola preparedness following an outbreak in the neighboring DRC in 2018 (26, 27). To combat the spread of Ebola, the Rwandan government implemented screenings for Ebola symptoms of people crossing the border between Rwanda and the DRC by the mass conducting of temperature checks and other preventive measures such as hand-washing, and public awareness campaigns. The government also prepared isolation units and testing centers in hospitals within 15 priority districts centers (27). The first COVID-19 case registered in Rwanda was on 2020-03-14, in the city of Kigali (28) and later spread to neighboring regions.

The government’s response to the COVID-19 pandemic in the initial stages was characterized by a proactive and aggressive approach. On 2020-03-21, the government announced the closure of all borders, and a nationwide lockdown, which involved strict measures such as the closure of all non-essential businesses and the suspension of public transportation (29). These were later replaced by introducing curfews, reducing capacity in public transportation and businesses, mandatory PCR testing upon arrival at Kigali International Airport as well as in-land borders, and temperature testing at the entrance of each shopping center.

In this work, we hypothesize that the spread of SARS-CoV-2 in Rwanda is connected to periods with higher population mobility, well represented by particular mobility recorded in transit stations. To test our hypothesis, we evaluated epidemiological trends in COVID-19 cases in relation to the change in population mobility in public transportation and other mobility categories. To our knowledge, this is the first study to explore transportation mobility dynamics in a low-income country during the COVID-19 pandemic.

## 2 Materials and methods

### 2.1 Public COVID data and governmental response (stringency index)

All newly confirmed COVID-19 cases per day and the stringency index were considered from 2020-03-14, the day when the first COVID-19 case was recorded in Rwanda, to 2022-10-15, the date when our analysis was performed. The data were retrieved from the open-access dataset developed by OWID: Our World in Data^1^, based on data collected from multiple national sources by the Center for Systems Science and Engineering at Johns Hopkins University. OWID has a spreadsheet with data from multiple countries. From the available data, we isolated the new cases, tests, vaccinations, and deaths for Rwanda. As the data are presented in daily updates, we aggregated them in 7 days blocks, representing weeks and removing seasonal variability in the data, such as weekends, backlog reporting, and holidays. We also extracted from this spreadsheet the stringency index values, which was provided by the Oxford COVID-19 Government Response Tracker (OxCGRT) developed by the Oxford Blavatnik School of Government. The index tracks how strict a government is in imposing restrictions. The index consists of a score ranging from 0 to 100, where 100 is the strictest level. To calculate the response of a particular government in restricting movement, a series of questions are considered with a score assigned to each. The questions are based on a series of restriction-oriented criteria, such as: (i) school closure; (ii) workplace closure; (iii) cancelation of public events; (iv) restriction on public gatherings; (v) closure of public transport; (vi) stay-at-home requirements; (vii) public information campaigns; (viii) restrictions on internal movements; (ix) restriction on the international travel; (x) intra provincial travel; (xi) inter provincial travel; (xii) curfew; (xiii) mandatory immunization for healthcare employees; (xiv) mandatory immunization for long-term care workers; (xv) mandatory immunization for public servants; and (xvi) mandatory immunization for K12 schools.

### 2.2 Mobility trends

To provide the scientific community with a real time measurement of people’s mobility trends, the tech company Google anonymously compiled the visits of their users to the Google Maps app locations categorized into: (i) retail and recreation; (ii) grocery and pharmacy; (iii) transit stations; (iv) workplaces; (v) residential, and (vi) parks. The public available data was recorded daily in 135 countries from March 03 2020 to October 15 2022.^2^ A pre-pandemic baseline value was established in the five-week period, from January 3 2020 to February 6 2020. To remove seasonal aspects of the data, we selected the records of Rwanda on a seven-day basis. Martinez and Kelvin (10) compared mobility data gathered from distinct tech companies and conclude the data is highly correlated.

### 2.3 Statistical analyses and figure generation

We displayed our data with graphs and figures using R Statistical Programming software version 4.2.2 (30). Graphs and figures were generated using ggplot2, ggally, plotly, gridExtra R packages. We tested for the correlation between variables using the Pearson test, which measures the strength and the direction of a linear relationship between two variables.
A cross-correlation analysis approach was also employed to examine the relationship between mobility at different locations and the COVID-19 cases. Utilizing time series cross-correlation allowed for a detailed analysis of the similarities between data points within a time series and the associated time lag.
Granger causality analysis was later used to test whether the past information on the change in the mobility can provide statistically significant information about the future change in the COVID-19 cases. Let us assume that for two stationary time series variables, X and Y, we want to test whether X Granger-cause Y; or in other words, we want to determine if past values of X are useful in predicting or forecasting the values of Y.
We first consider a restricted model which is a univariate Autoregressive (AR) model of Y. The equation will be:

$$Y_{t}=\overset{k}{\underset{n=1}{\sum }}A_{n}Y_{t-n}+\varepsilon_{t}$$

Where $A_{n}$ represents the AR model coefficients and k the order of the AR model.
Secondly the unrestricted model which is a bivariate autoregressive model:

$$Y_{t}=\overset{k}{\underset{i=1}{\sum }}A_{i}Y_{t-i}+\overset{k}{\underset{i=1}{\sum }}B_{i}X_{t-i}+\varepsilon_{t}$$

To test if X granger cause Y, we used the Wald test which follows a chi-square distribution, where the test will compare the performance of the restricted model against the unrestricted model.
$H_{0}$ : $B_{1}=B_{2}=\ldots =B_{k.}$
$H_{1}$ : At least one of the $B_{1}=B_{2}=\ldots =B_{k}\neq 0$
We reject the null hypothesis when the calculated value of p is less than the level of the significance level α=0.05 , and conclude that X granger cause Y.

## 3 Results

### 3.1 Epidemiological characteristics of COVID-19

**Figure 1:**
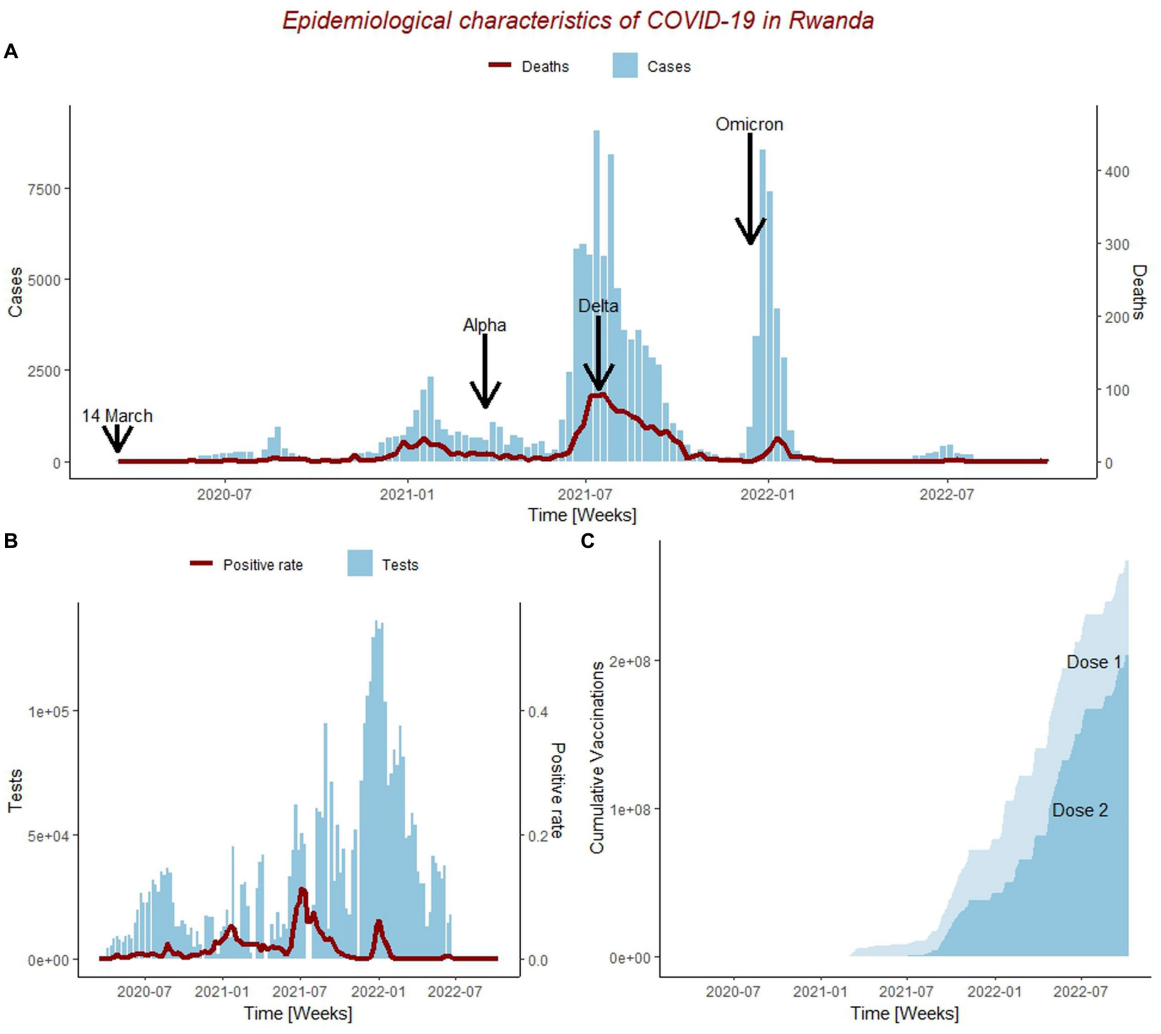
Epidemiological characteristics of COVID-19 outbreak in Rwanda from 14/03/2020 to 15/10/2022. **(A)** Epidemiological curve for weekly new COVID-19 cases (blue) and deaths (red); Including the date when the first case was ever reported in Rwanda on for the Wuhan strain (14/04/2020), Alpha, Delta, and Omicron Variants of Concern periods. **(B)** Trend of weekly total number of the people tested for COVID-19 (blue) and positive rate (red). **(C)** Cumulative number of COVID-19 vaccine doses administered; Dose1 represents the cumulative number of people who received at least one dose of COVID-19 vaccine, and Dose2 represents the cumulative number of people who received at least two doses of COVID-19 vaccine.

The first COVID-19 case in Rwanda was reported on 2020-02-14 while the first death was reported on 2020-05-25. Up to the week of 2022-10-15, 132,551 COVID-19 cases and 1,467 deaths had been recorded in Rwanda (Figure 1A). The majority of cases (80%) occurred in individuals over the age of fifty. On average, approximately 1,000 PCR tests were conducted per day, with a COVID-19 recovery rate of 98% (31). We further divided the epidemiological curve of COVID-19 in Rwanda according to variants of concern (VoCs). The first Alpha variant (B.1.1.7) case was reported on 2021-03-21, the first Delta (B.1.617.2) case was reported on 2021-07-14, and the first Omicron (B.1.1.529) was reported on 2021-12-14. The highest peak in the total number of infections and deaths were recorded during the Delta variant period, followed by the Omicron and Alpha variant periods. The Delta variant wave persisted longer than the Omicron and Alpha waves, respectively. Next, in Figure 1B, we show the number of tests and test positivity rate in Rwanda. The highest volume of tests was performed during the week of 2021-12-27, 2 weeks after the first Omicron case, while the highest test positivity recorded was during the Delta VoC period, with a rate of 0.114. Finally, in Figure 1C, we show the vaccination coverage in Rwanda. The first vaccine was administered on 2021-03-01, and more than 60% of the population was fully vaccinated (two or more doses) by 2022-10-15.

### 3.2 Impact of restrictive measures on mobility

**Figure 2:**
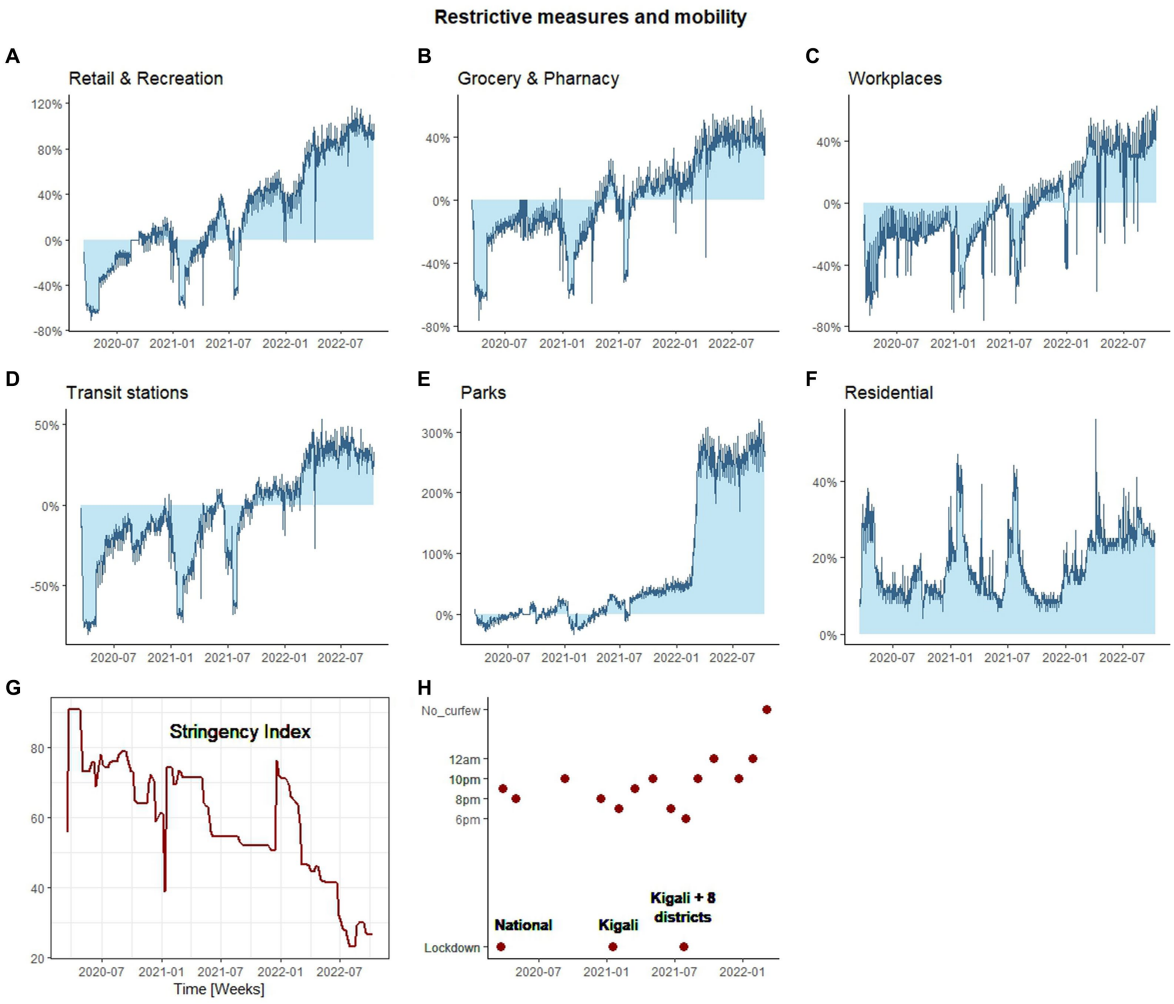
The impact of COVID-19 restrictive measures on the mobility flow. The change in mobility flow during the COVID-19 outbreak at different locations. **(A)** Retail and recreation, **(B)** Grocery and Pharmacy, **(C)** Workplaces, **(D)** Transit stations, **(E)** Parks, **(F)** Residential. The change in mobility is in percentage where 0% represent no change in mobility flow compared to the baseline. The baseline represents the mobility flow for the pre-pandemic period from 3 January 2020 to 6 February 2020. **(G)** Represents the change in the stringency index during COVID-19, **(H)** represents curfew restrictions imposed during COVID-19 outbreak.

The impact of governmental restrictions, such as lockdowns and curfews in controlling the population flow of mobility was analyzed (Figure 2). We compiled the categorized mobility trends in Rwanda from 2020-03-03 to 2022-10-15 to depict movement patterns of the population during the COVID-19 pandemic.

First, we report that during the onset of COVID-19 in Rwanda, the mobility of people in retail and recreation (Figure 2A), grocery and pharmacy (Figure 2B), workplace (Figure 2C), and transit stations (Figure 2D) sharply decreased compared to the baseline (−63.45, −60.71%, −54.86, −75%). Moreover, movement in Parks (Figure 2E) decreased to a lower level (−19%), whereas stay-in-house mobility (i.e., residential; Figure 2F) increased by 30.43% in the same period. These declines coincided with the implementation of stringent governmental restrictions, including a national lockdown, resulting in the highest recorded stringency index of 90.74 (Figure 2G). Similar patterns of reduced mobility in outdoor activities compared to the baseline, were observed in early and mid-2021, during the subsequent lockdown periods (Figure 2H). However, in the period when these subsequent lockdowns were implemented, the stringency index did not increase sharply, as it happened during the onset of the COVID-19 pandemic when the national lockdown was implemented (Figure 2G). In early 2021, there was a reduction of more than 50% in outdoor activity mobility compared to the baseline. Interestingly, the stringency index concurrently declined to 38.9, which presents a paradox. However, it is important to highlight that only Kigali was subjected lockdown during this period, while the movement in the rest of the country was allowed with curfew restrictions. A similar situation was observed in mid-2021, where only Kigali city and eight other districts (Burera, Gicumbi, Kamonyi, Musanze, Nyagatare, Rubavu, Rwamagana, and Rutsiro) out of the total 30 districts were subjected to lockdown.

As the lockdown measures gradually eased, curfew hours extended, and vaccination efforts intensified, there was a partial recovery in mobility. However, mobility flow at these four outdoor categories remained below the baseline for the entirety of 2020 and midway through 2021. In contrast, mobility flow in parks increased above the baseline with two small peaks, indicating that parks were visited more frequently than during pre-pandemic period. Midway through 2021 and on, small spikes in increasing mobility above the baseline were observed in retail and recreation, grocery and pharmacy, and transit stations. After this period, outdoor activities were consistently recorded above the baseline, and mobility was higher to pre-pandemic stages.

### 3.3 How COVID-19 cases were associated with mobility

The examination of cross-correlations between mobility patterns and COVID-19 cases, as presented in Table 1, unveiled distinct temporal connections. The type of mobility most strongly correlated with COVID-19 cases was park mobility, demonstrating a robust correlation of 0.596 at a lag of 3 weeks. Following closely was grocery and pharmacy mobility, showing a correlation of 0.503 at a lag of 2 weeks, then workplaces mobility with a correlation at 0.451 with a lag of 3 weeks, and retails and recreation mobility, with a correlation of 0.446 at a lag of 3 weeks. The correlation pattern suggests that increased mobility in these locations might precede an uptick in COVID-19 cases. Transit station mobility displayed the lowest correlation magnitude of 0.386 compared to other mobility types, and the lengthiest lag of 9 weeks. Notably, the residential mobility demonstrated a weak negative correlation compared to other mobilities of−0.105 at a lag of 2 weeks, hinting at a subtle inverse relationship between heightened residential mobility and a minor reduction in COVID-19 cases after a two-week period, suggesting that changes in human mobility is correlated with the spread of COVID-19 virus with different time lags in different locations.

**Table 1:** Cross correlation between COVID-19 cases and mobility patterns in Rwanda.

Cross-correlation											
	Lags										
	0	1	2	3	4	5	6	7	8	9	10
Transit stations	−0.294	−0.28	−0.21	−0.088	0	0.123	0.249	0.335	0.38	**0.386**	0.326
Retail & recreation	0.339	0.349	0.423	**0.446**	0.389	0.262	0.162	0.076	0.021	−0.064	−0.112
Grocery & pharmacy	0.405	0.435	**0.503**	0.499	0.427	0.307	0.233	0.166	0.109	−0.001	−0.061
Parks	0.39	0.452	0.554	**0.596**	0.548	0.471	0.438	0.414	0.366	0.295	0.226
Workplaces	0.361	0.362	0.414	**0.451**	0.401	0.273	0.152	0.031	−0.05	−0.152	−0.202
Residential	−0.074	−0.07	**−0.11**	−0.095	−0.039	0.094	0.202	0.29	0.324	0.397	0.415

Bold values represent the highest cross-correlation values.

To assess the potential Granger causation of variable X (mobility) on variable Y (COVID-19 cases), the Granger causality test was utilized, preceded by an examination of the Granger causality test assumption, which is stationarity of the time series data. Augmented Dickey Fuller (ADF) test was used to check whether the time series are stationary or not. The results of this ADF test revealed non-stationarity for all the mobility variables and COVID-19 cases variables. To rectify this, the variables were transformed using differences, resulting in the achievement of stationarity. Subsequently, the Granger causality test was applied to the transformed variables, and the resulted *p*-values are presented in Table 2, as well as different time lag values.

**Table 2:** Granger causality test.

*p*-Value				
Mobility	Lags			
	1	2	3	4
Transit stations	0.03765	**0.01904**	0.02357	0.1023
Retail & recreation	0.001909	0.001441	**0.001073**	0.006655
Grocery & pharmacy	0.03132	**0.01673**	0.01933	0.07578
Parks	**0.0007394**	0.6116		
Workplaces	0.0001017	**0.0000094**	0.000132	0.0004146
Resi dential	0.01351	**0.01095**	0.01663	0.05606

Bold values represent the highest *p*-values.

The *p*-values represented in Table 2, show that mobility in transit stations, grocery and pharmacy, workplaces, and residential were highly significant for the lag of 2, indicating that alterations in transit stations, grocery and pharmacy, workplaces, and residential mobility influence changes in COVID-19 cases specifically with the lag of 2 weeks, whereas retail and recreation was highly significant at the lag of 3, but parks mobility at the lag of 1.

**Figure 3:**
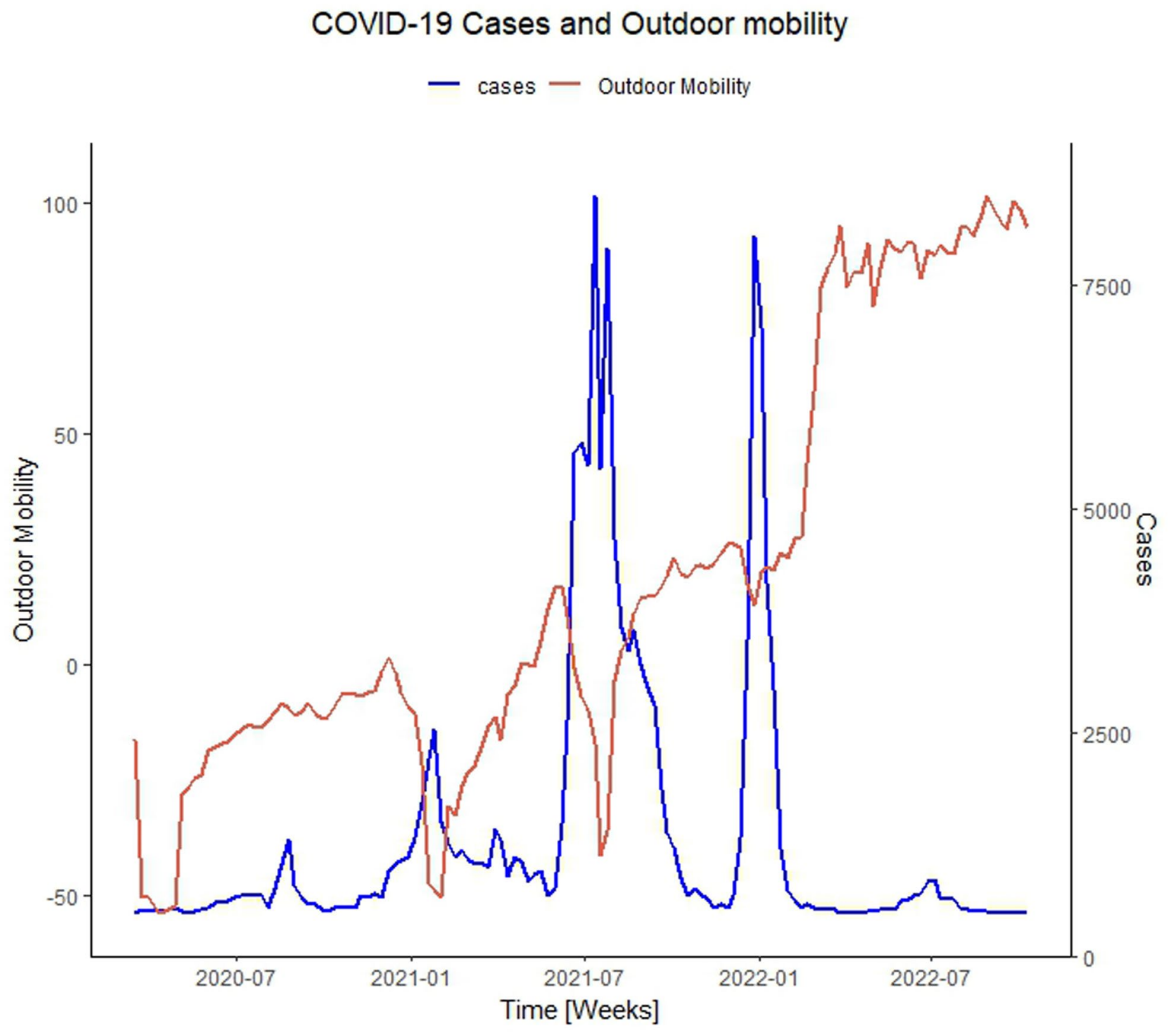
The relationship between the outdoor mobility trend and COVID-19 cases. This figure illustrates the relationship between the outdoor mobility flow (red line) and the epidemiological trend for COVID-19 cases (blue line).

To analyze in a temporal perspective the relation between the overall outdoor mobility and COVID-19 cases, Figure 3 is provided. First, we report that during the period in which outdoor mobility was at its lowest (2020-04-06), COVID-19 cases followed a similar trend, where only 125 total cases were reported in Rwanda. At this time, a national lockdown and a curfew of 9 PM were implemented. The largest spike in cases occurred during the onset of the Delta variant in July 2021. Prior to this spike in cases, there was a notable increase in transit station mobility, transitioning from a decline of−53.68% below the baseline in the week of 2021-04-13, to an increase of−8.11% above the baseline in the week of 2021-08-17. This rise in outdoor mobility also preceded other spikes in cases occurred during Alpha and Omicron VoC periods. The first peak in cases occurred on the Week of 2020-08-24, with 931 cases reported merely 1 week after a spike in outdoor mobility in the Week of 2020-08-17. A subsequent peak in cases occurred during the Week of 2021-01-25, with 2,329 cases, preceded by an increase in outdoor mobility 7 weeks earlier in the Week of 2020-12-07. Another peak emerged in the Week of 2021-07-12, with 9,078 reported cases, nearly 6 weeks after a rise in outdoor mobility was observed during the Week of 2021-05-31. This suggests a link between increased outdoor mobility flow and higher number of infections. As cases started to increase, curfews and limited public transport had been mandated, resulting in the reduction of outdoor mobility. Based on these observations, we propose that the spike in Delta cases reported in Rwanda may have been partially attributed to the alleviated social restrictions, reflected in the lower stringency index score. This relaxation of restrictions likely encouraged the flow in outdoor mobility, thereby contributing to a higher number of infections due to increased social contact.

## 4 Discussion and conclusion

In our study, we found that the transmission of COVID-19 in Rwanda is generally preceded by periods of higher population mobility with particular focus on public transport mobility, which is known to play a detrimental role in the life of the working class of Rwanda. This study shed light on several key aspects related to this impact of public transportation on the transmission of COVID-19 in Rwanda. The study reveals how the population responded to restrictive measures implemented by the government in reducing the population mobility; mobility patterns in retail and recreation, grocery and pharmacy, workplace, and transit stations exhibited a decrease at the onset of the pandemic, on early 2012, and mid-2021, when lockdowns were implemented (national lockdown, lockdown in Kigali only, and lockdown in Kigali and other 8 districts of the country). The mobility in parks; however, remained consistent throughout the pandemic compared to other activities. This was likely attributed to tourism activities remaining open during the pandemic period. A study conducted on a global scale have shown that mobility in outdoor environments in 153 countries is not associated to the increase in COVID-19 transmission (32). During the early stages of the pandemic, when strict measures were implemented, mobility in public transportation reached its lowest point. The study also demonstrated that the national lockdown and curfew measures were associated with a considerable change in the stringency index and population mobility. This finding underscores the influence of curfews on transportation patterns and highlights their effectiveness in controlling mobility. Some studies have found that imposing curfew was effective in altering the spread of COVID-19 epidemic (33–35). This study also reveals the significant impact of public transportation on the movement of people within the country. It was evident that the mobility in transit stations was strongly correlated with mobility in other activities, highlighting the dependence of Rwanda on its public transportation system.

Further investigation into the association between transit station mobility and COVID-19 cases demonstrated interesting insights. The epidemiological transmission of COVID-19 in Rwanda revealed a significant number of cases and deaths, with the highest numbers recorded and largest spikes in cases during the Delta variant period, which was similarly observed in South Africa (36) and Africa on a continental level (37), however, this was not the case in Canada (10) and Europe on a continental level (37), where the cases peaked during successive waves of Omicron and its sub-lineages (B.1.1.529, BA.4/0.5, XBB.1.5).

The spike in cases during the Alpha and Delta VoC periods in Rwanda were preceded by a notable surge in outdoor mobility (Figure 3); however, immediately after cases started rising during these VoC periods, stricter restrictions were implemented resulting in a reduction of mobility in outdoor places for more than 50% below the baseline. This suggests that the relaxation of social restrictions resulted in the subsequent increase in outdoor mobility flow, which may have contributed to a higher number of infections. These findings align with previous studies that have consistently demonstrated the role of human mobility and transportation in facilitating the spread of COVID-19 and demonstrate a positive correlation between mobility patterns, such as visits to non-essential businesses and public transportation usage, and increased infection rates in the US (7), China (6), and Kenya (9).

This study also demonstrated through granger causality test that mobility in transit stations, grocery and pharmacy, workplaces, and residential were highly significant for the lag of 2, indicating that alterations in transit stations, grocery and pharmacy, workplaces, and residential mobility influence changes in COVID-19 cases specifically with the lag of 2 weeks, whereas retail and recreation was highly significant at the lag of 3, but parks mobility at the lag of 1. The study shed light on how mobility variables are important in predicting future trends or changes in COVID-19 cases. However, it’s important to acknowledge that there may be underlying factors influencing the observed trends and changes in COVID-19 cases unexplored in our study due to the lack of data, therefore, this prevents us from making definitive causal statements regarding the relationship between mobility, particularly in public transportation, and COVID-19 transmission. Nonetheless, our aim was to emphasize the predictive value of mobility variables, particularly in public transportation, in anticipating future trends in COVID-19 cases. While our findings are preliminary, they serve as valuable insights and could be utilized as significant explanatory factors in the development of multivariate models alongside other variables.

It is crucial to acknowledge the limitations of this study. This research establishes a link between mobility in public transportation and COVID-19 transmission in Rwanda; however, there may be other underlying factors not fully explored. In addition, for population mobility, future studies may explore those factors that could correlate with the number of infected cases, such as air pollution, weather seasonality, and cultural behaviors which can influence the social interaction of the population, closeness in proximity among passengers, and inadequate ventilation among others.

This research establishes a link between mobility in public transportation and COVID-19 transmission in Rwanda; however, there may be other underlying factors not fully explored. This research was also limited in terms of data availability. Google mobility data results in data gaps on the days and/or location where there is not enough data to anonymously estimate the change of mobility from the baseline. This resulted in mobility data from Rwanda having some gaps for regions other than Kigali. Consequently, our ability to assess the representativeness of our study population is limited. Therefore, these findings should be interpreted with caution as the mobility of people in Kigali, the most urban city in Rwanda, may not be generalized to the patterns of people in other areas of the country.

This study aimed to evaluate epidemiological trends in COVID-19 cases in relation to the change in population mobility in public transportation. In this study, we hypothesized that the spread of SARS-CoV-2 and the use of public transportation in Rwanda are closely connected. The findings of this study show that mobility in transit stations was strongly correlated with mobility in other activities, highlighting the dependence of Rwanda on its public transportation system. This study also found that spikes in cases were preceded by an increase in transit station mobility; and through Granger causality analyses, the study revealed that past information on the change in mobility in public transportation can significantly predict the change in COVID-19 cases at the lag of 2 weeks.

This research provides valuable insights that can inform public health strategists regarding appropriate disease control interventions in Rwanda and suggests the need for continued vigilance and measures to regulate public transportation during infectious disease outbreaks such as promoting contactless payment options, use of natural and systems of ventilation, hygiene and disinfection at the entrance of the stations and busses, and restricting the number of passengers among others.

## Data availability statement

The original contributions presented in the study are included in the article/supplementary material, further inquiries can be directed to the corresponding author.

## Author contributions

BU: Conceptualization, Data curation, Formal analysis, Investigation, Methodology, Software, Visualization, Writing – original draft, Writing – review & editing. JT: Formal analysis, Visualization, Writing – review & editing. BH: Data curation, Formal analysis, Investigation, Visualization, Writing – review & editing. JU: Formal analysis, Validation, Visualization, Writing – review & editing. PN: Formal analysis, Validation, Visualization, Writing – review & editing. DK: Conceptualization, Data curation, Formal analysis, Funding acquisition, Investigation, Methodology, Project administration, Resources, Software, Supervision, Validation, Visualization, Writing – original draft, Writing – review & editing. GS: Conceptualization, Data curation, Formal analysis, Methodology, Software, Visualization, Writing – original draft, Writing – review & editing.

## Footnotes

^1^https://ourworldindata.org/coronavirus

^2^https://www.google.com/covid19/mobility/
